# Investigation of two different human d-dimer assays in the horse

**DOI:** 10.1186/s12917-022-03313-5

**Published:** 2022-06-15

**Authors:** Marie Louise Honoré, Tina H. Pihl, Tanne M. Busk-Anderson, Laura L. Flintrup, Lise N. Nielsen

**Affiliations:** 1 Department of Veterinary Clinical Sciences, Faculty of Health and Medical Sciences (SUND), University of Copenhagen, Hoejbakkegaard Allé 5a, 2630 Taastrup, Denmark; 2 Section for Internal Medicine, Oncology and Clinical Pathology, Faculty of Health and Medical Sciences (SUND), University of Copenhagen, Dyrlaegevej 16, 1870 Frederiksberg C, Denmark

**Keywords:** Fibrin degradation products, Equine, Hemostasis, Hypercoagulation, Thrombosis, Validation

## Abstract

**Background:**

D-dimer has value as a marker of thrombosis in critically ill horses and can provide additional information about prognosis. However, there are currently no equine species-specific d-dimer assays available, nor has there been any formal investigation of the applicability of human d-dimer assays in horses, so it is unknown, which assay performs best in this species. The aim of this study was therefore to evaluate and compare two human d-dimer assays for their applicability in horses.

The study included four groups of horses: clinically healthy horses, horses with gastrointestinal (GI) disease and mild systemic inflammation based on low serum amyloid A (SAA) (low SAA group), horses with GI disease and strong systemic inflammation based on high SAA (high SAA group) and, horses with thrombotic GI disease caused by *Strongylus vulgaris* (also called non-strangulating intestinal infarction (NSII)) (NSII group). The assays evaluated were the STAGO STA-Liatest D-di + (Stago) and NycoCard™ D-dimer (NycoCard). Intra- and inter-coefficients of variation (CV) were assessed on two d-dimer concentrations, and linearity under dilution was evaluated. A group comparison was performed for both assays across the four groups of horses. A Spaghetti plot, Spearman Correlation, Passing Bablok regression and Bland–Altman plot were used to compare methods in terms of agreement.

**Results:**

Ten horses were included in the clinically healthy group, eight in the low SAA group, eight in the high SAA group, and seven in the NSII group. For the Stago assay, intra- and inter-CVs were below the accepted level except for one inter-CV. The NycoCard assay did not meet the accepted level for any of the CVs. The linearity under dilution was acceptable for both the Stago and NycoCard. In the group comparison, both methods detected a significantly higher d-dimer concentration in the high SAA and NSII groups compared to the clinically healthy group. Method agreement showed slightly higher d-dimer concentrations with NycoCard compared to Stago. The overall agreement was stronger for the lower d-dimer concentrations.

**Conclusion:**

Both the Stago and the NycoCard were found to be applicable for use in horses but were not directly comparable.

**Supplementary Information:**

The online version contains supplementary material available at 10.1186/s12917-022-03313-5.

## Background

Making a definitive diagnosis of thrombosis in equine medicine is often challenging as there, to the best of the authors knowledge, currently is no established gold standard for use in equine clinical practice. In humans, a definitive diagnosis of thrombosis relies on different imaging modalities such as contrast venography and different ultrasonographic techniques [1–4]. It is often not practical to use these techniques in horses in a clinical setting due to the size of the animal and the potential need for general anesthesia.

D-dimer represents a good surrogate biomarker of thrombosis. It is the end product of fibrinolysis and can only be measured in plasma after plasmin degradation of cross-linked fibrin [5, 6]. It is thus a marker of the fibrinolytic system [7]. A low d-dimer concentration is an effective rule-out marker for thrombosis, while a high concentration is indicative of, but cannot confirm thrombosis [8, 9]. Due to the lack of a species-specific assay, d-dimer analyzes in equine medicine have been performed using different human assays [10–17]. To the best of the authors’ knowledge, there has not been a full investigation into the applicability of these assays in horses and thus, it is currently uncertain, which assay performs most advantageously in this species [10]. Plasma d-dimer has been examined in horses with diseases predisposing for concurrent hemostatic imbalances. In particular, horses with gastrointestinal (GI) disease and a strong systemic inflammatory response may develop hemostatic aberrations [15, 16, 18]. D-dimer measurements at admission and during hospitalization can be used to monitor the improvement in medical and surgical colic patients [16], and function as a prognostic marker of survival [15, 18]. The NycoCard™ D-dimer assay (NycoCard) has been used in horses with severe GI disease. An increased d-dimer concentration was found in horses with surgical colic [12], and d-dimer was found to be a valuable test for diagnosing disseminated intravascular coagulation (DIC) [10].

The parasite *Strongylus vulgaris* (*S. vulgaris*) migrates in the mesenteric arteries, causing thrombosis [19–22] that can lead to non-strangulating intestinal infarctions (NSII) [13]. The STAGO STA-Liatest D-di + (Stago) assay has been used to show that foals with migrating *S. vulgaris* larvae have an increased d-dimer concentration that correlates with the number of larvae in the cranial mesenteric artery (CMA) [23]. However, no studies have evaluated d-dimer concentrations in adult horses with NSII.

Due to the lack of a formal investigation into the applicability of the Stago and NycoCard assays in equine medicine, the aim of this study was to evaluate and compare these two human d-dimer assays in relation to their application in horses.

## Results

### Horses

The clinically healthy group consisted of ten horses (eight mares and two geldings), with a mean age of 11.2 years (min. 4 – max. 22.8 years), and a mean bodyweight (BW) of 559.5 kg (min. 411 – max. 687 kg). Breed distribution was as follows: seven Standardbreds, two warmbloods and one of unknown breed. The low SAA group included eight horses with GI disease and a mild systemic inflammatory response with SAA between 30–100 mg/L at admission. This group consisted of five mares and three geldings, with a mean age of 11.8 (min. 4 – max. 23.2 years), and a mean BW of 404.3 kg (min. 286 – max. 700 kg). Breed distribution was as follows: three Icelandic horses, one Clydesdale, three ponies, and one of unknown breed. The high SAA group included eight horses with GI disease and a strong systemic inflammatory response with SAA > 1,000 mg/L at admission. This group consisted of two mares, five geldings, and one stallion with a mean age of 13.4 years (min. 3.1 – max. 24.1 years) and a mean BW of 529.8 kg (min. 350 – max. 666 kg). Breed distribution was as follows: two Icelandic horses, one pony, four warmbloods, and one of unknown breed. The NSII group included seven horses with NSII: four mares and three geldings with a mean age of 11.8 years (min. 9 – max. 17.4 years) a mean BW of 446.8 kg (min. 361 – max. 540 kg). Breed distribution was four warmbloods and three ponies.

### Sample handling

Samples for two out of the seven horses in the NSII group were collected in 3.8% (rather than 3.2%) sodium citrate tubes.

### Test evaluation

The intra- and inter-CVs for the Stago assay were below the accepted level of 5%, with the exception of the inter-CV for the low pool, whereas the intra- and inter-CVs for the NycoCard were above the accepted level of 10% in all four CVs (Table 1).

**Table 1:** Imprecision study for the STAGO STA-Liatest D-di + (Stago) and NycoCard.™ D-dimer (NycoCard) assays. Intra- and inter-coefficients of variation (CV) for the low and medium pool for the Stago and NycoCard were calculated as CV % = (standard deviation / mean) * 100

	Stago	NycoCard
Intra-CV % Low pool	4.66% (0.23; 0.01)	35.14% (0.15; 0.053)
Inter-CV % Low pool	5.98% (0.21; 0.01)	33.99% (0.13; 0.04)
Intra-CV % Medium pool	1.79% (0.51; 0.01)	11.11% (0.60; 0.07)
Inter-CV % Medium pool	2.63% (0.49; 0.01)	12.33% (0.58; 0.07)

(mean; standard deviation)

**Fig. 1:**
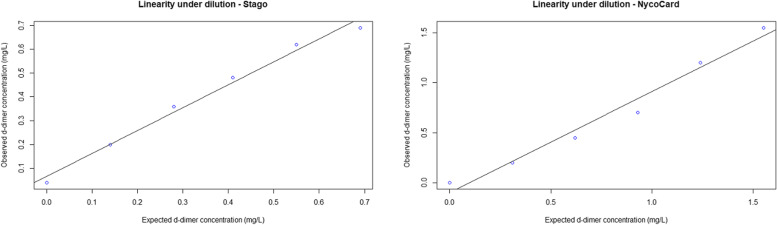
Linearity under dilution for the STAGO STA-Liatest D-di + (Stago) and NycoCard.™ D-dimer (NycoCard) assays using a Passing Bablok regression. A slope of 1.00 (95% confidence interval (CI) 0.75–1.14) and a Y-intercept of 0.065 (95% CI 0.04–0.17) were found for the Stago, while a slope of 1.00 (95% CI 0.73–1.37) and Y-intercept of -0.075 (95% CI -0.58–0.00) were found for NycoCard. Note the different axes in the two graphs

The results from the linearity under dilution showed agreement between the observed and expected values of between 89 and 70% for the Stago and 97% and 65% for the NycoCard assay. The linearity under dilution for NycoCard showed a slope of 1.00 (95% Confidence interval (CI) 0.73–1.37) and Y-intercept of -0.075 (95% CI -0.58–0.00). For the Stago, a slope of 1.00 (95% CI 0.75–1.14) and Y-intercept of 0.065 (95% CI 0.04–0.17) were found (Fig. 1).

### Group comparison

**Fig. 2:**
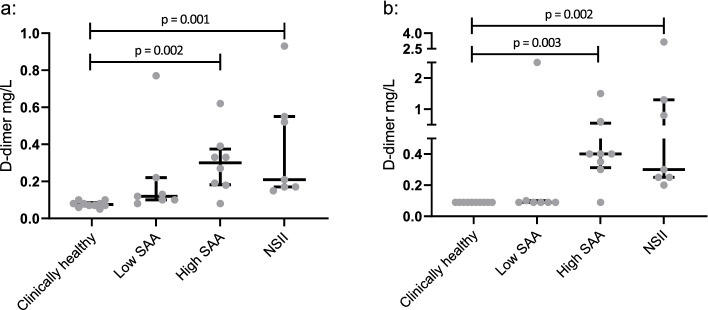
Group comparison across the four groups of horses by the STAGO STA-Liatest D-di + (Stago) and NycoCard.™ D-dimer (NycoCard) assays. **a** For the Stago assay, a significant difference was seen between the clinically healthy group, the high serum amyloid A (SAA) group and the non-strangulating intestinal infarctions (NSII) group. **b** For the NycoCard assay, a significant difference was seen between the clinically healthy group, the high SAA group, and the NSII group. The highest value in the low SAA group for the Stago and the highest value in the low SAA group for the NycoCard are not the same horse. Horizontal lines represent median and interquartile range. Note the different y-axes in the two graphs

The group comparison showed a statistically significant difference between the groups for both assays (*p* = 0.0003). For the Stago assay, the post hoc analysis showed a significantly higher d-dimer concentration in the high SAA group (median 0.3 mg/L, 1^st^ and 3^rd^ quartile (Q_1_-Q_3_): 0.18–0.38) (*p* = 0.002) and in the NSII group (median 0.21 mg/L, Q_1_-Q_3_: 0.17–0.55) (*p* = 0.001) compared to the clinically healthy group (median 0.075 mg/L, Q_1_-Q_3_: 0.07–0.09). For the NycoCard assay, the post hoc analysis showed a significantly higher d-dimer concentration in the high SAA group (median 0.4 mg/L, Q_1_-Q_3_: 0.31–0.55) (*p* = 0.003) and in the NSII group (median 0.3 mg/L, Q_1_-Q_3_: 0.25–1.3) (*p* = 0.002) compared to the clinically healthy group (median < 0.1 mg/L, Q_1_-Q_3_: < 0.1- < 0.1) (Fig. 2).

One horse from the low SAA group was excluded based on Cook’s distance. The horse had a d-dimer concentration of 9.38 mg/L as measured by the Stago assay. For completeness, this horse was also included in the group comparison. The only change was that a significant difference was then also seen between the clinically healthy group and the low SAA group (*p* = 0.04).

### Method comparison

**Fig. 3:**
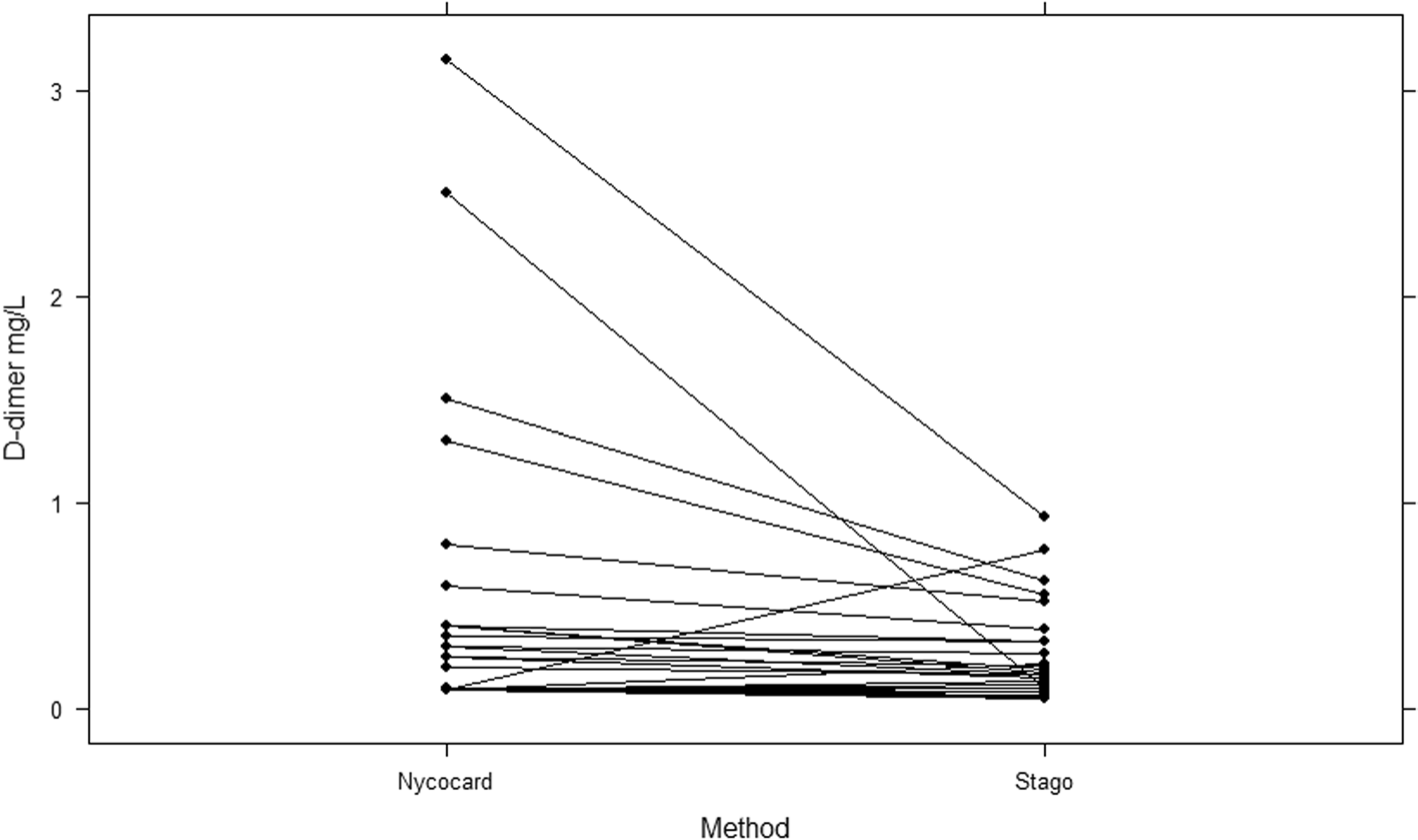
Spaghetti plot displaying the subject-wise trends for the d-dimer (mg/L) concentration for each horse measured with STAGO STA-Liatest D-di + (Stago) and NycoCard.™ D-dimer (NycoCard) assays. NycoCard measurements were generally higher than the Stago measurements, but there was relatively good agreement between the two assays, especially for the lower concentrations. Three horses had markedly varying results: two were markedly higher with the NycoCard assay, and one was markedly higher with the Stago assay

A spaghetti plot showed that d-dimer measurements from the NycoCard assay were generally higher than Stago measurements, but agreement between the two assays seems to be good, especially for the lower d-dimer concentrations. Three horses had noticeably different measurements across the two assays. Two horses had a markedly higher d-dimer concentration with the NycoCard compared to the Stago assay, whereas one horse had a markedly higher d-dimer concentration with the Stago compared to the NycoCard assay (Fig. 3).

A Passing Bablok regression between the Stago and NycoCard measurements gave a slope of 1.61 (95% CI: 1.02–2.61) with an intercept of -0.04 (95% CI: -0.14–0.02). The Spearman correlation between the Stago and the NycoCard assays showed a significantly (*p* < 0.0001) positive correlation with an r-value of 0.7 (95% CI: 0.46–0.85).

**Fig. 4:**
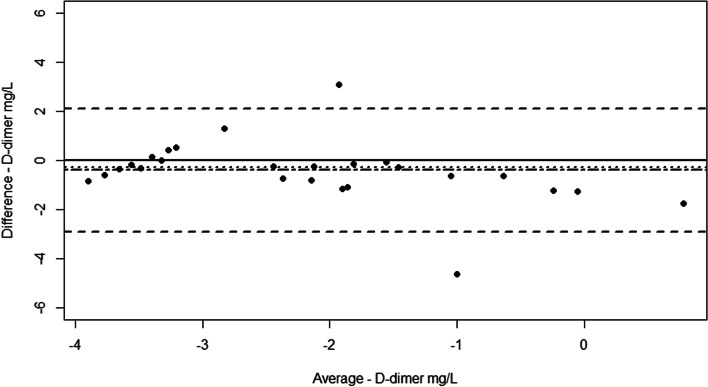
Bland–Altman plot displaying the difference plotted against the average of log2 transformed STAGO STA-Liatest D-di + (Stago) and NycoCard.™ D-dimer (NycoCard) assay measurements including 95% limits of agreement (LOA). Mean -0.4 (dot-dashed line), median -0.3 (dotted line), and 95% LOA -2.9–2.11 (dashed lines)

A Bland–Altman plot showed that all but two measurements were within the 95% limits of agreement of -2.9–2.11 mg/L (log2) with a mean of -0.4 mg/L (log2) and a median of -0.3 mg/L (log2) (Fig. 4).

## Discussion

Both assays were able to measure equine d-dimer in PPP and performed acceptably in terms of reliability and validity. They found a significantly higher d-dimer concentration in the high SAA and NSII groups compared to the clinically healthy horses and showed good agreement in terms of classifying horses as having a low or high d-dimer concentration.

The NycoCard assay did not meet the accepted intra- and inter-CV limits of 10% for manual assays [24]. The deviation was most substantial in the low pool (Table 1). One explanation for this could be that the assay is relatively crude when displaying results showing only a single decimal. The d-dimer concentrations in the low pool varied from 0.1 to 0.2 mg/L, which is a 100% increase, thereby resulting in a large variability between measurements. In the medium pool, the d-dimer concentrations varied from 0.5 to 0.7 mg/L, which is a proportionally smaller increase, thus resulting in CVs that were much closer to the accepted limit. In addition, the low d-dimer concentrations of 0.1 and 0.2 mg/L are likely to be of minimal clinical relevance as they historically are considered within normal reference intervals when measured using alternative d-dimer assays [15]. The Stago assay met the accepted limit of 5% for intra- and inter-CVs [24], with the exception of the low pool, which had an inter-CV of 5.98%. This is a small deviation from the intended precision level and perhaps repeating measurements more than 10 times would have given an inter-CV below 5% [24]. Furthermore, as with the NycoCard assay, results regarding the low pool are of less clinical relevance, so a slightly larger variation can be tolerated [15]. Regardless of the inter- and intra-CV values, both assays were still considered to be of clinical relevance as often the most important thing in a clinical setting is to know that horses are classified correctly in terms of having a low or high d-dimer concentration, and not necessarily, what the exact concentration is.

The agreement between the expected and obtained values for the Stago assay varied between 89 and 70%, with the highest agreement in the 80% pool and the lowest agreement in the 20% pool. For the NycoCard assay, the agreement varied between 97 and 65% for the same pools. Agreement should ideally be 100% but is usually between 80 to 120% [24]. For the Stago assay, the low agreement seen in the pool with the low d-dimer concentration could possibly be explained by the d-dimer concentration being below the preset detection limit for the assay, which increases the inaccuracy of the measurements. For the NycoCard assay, the similar low agreement in the 20% pool could be explained by the measuring increments of 0.1 mg/L, which has a substantially larger influence in low d-dimer concentrations. The Passing Bablok regression showed a good coherence between the obtained and expected d-dimer concentrations for both assays. For the Stago assay, the 95% CI for the slope contained 1 and the CI for the Y-intercept was very close to containing 0. For the NycoCard assay, the 95% CI for the slope contained 1 and for the Y-intercept contained 0, which are considered optimal values for a 100% agreement between expected and observed values [24].

Due to the lack of an equine species-specific recombinant d-dimer antibody, the linearity under dilution was investigated using a heterologous d-dimer antibody. A previous study [25] found that the fibrinogen molecule is highly conserved between species and that there is extensive amino-acid homology between specific human and equine fibrinogen sequences. However, whether the breakdown products of cross-linked fibrin are identical in horses and humans remains unknown. The analyzed assays are heterologous, and we do not have any available equine d-dimer that can be added and assessed in terms of recovery. As the human assays resulted in d-dimer concentrations within the expected range for the four different groups of horses, we choose to interpret that the assays could in fact measure equine d-dimer.

The linearity under dilution was investigated on a d-dimer pool with a d-dimer concentration of 0.72 mg/L. This was the highest pool it was possible to create in this study. It could be argued that a high pool with even higher values, as found in the literature [15], would provide a better view of how precisely the two assays can measure the higher d-dimer concentrations.

In the group comparison, both assays measured a significantly increased d-dimer concentration in the high SAA and NSII groups compared to the clinically healthy group. However, no significant difference was seen across the three different disease groups. This indicates that d-dimer increases in horses with GI disease and systemic inflammation, as well as in horses with GI disease and thrombosis. It was not possible to differentiate between horses with inflammation and NSII in this study. In comparison, increased d-dimer concentrations have been found in dogs with systemic inflammation, rendering d-dimer a good rule-out marker for thrombosis, though not necessarily a good rule-in marker [26, 27].

One horse in the low SAA group had a markedly increased d-dimer concentration measured by the Stago assay (9.38 mg/L), while it had a d-dimer concentration of < 0.1 mg/L when measured by the NycoCard assay. The sample from this horse was not collected or handled any differently than the samples from the other horses. The measured fibrinogen concentration was within normal limits. The horse was intra-operatively diagnosed with peritonitis of unknown origin, which might explain why it would have an elevated d-dimer concentration compared to clinically healthy horses. However a d-dimer concentration of around 9 mg/L seems improbably high compared to previous studies [15, 18], and none of the other horses in the study—even those that were more severely ill—had d-dimer concentrations that came close to this concentration. The d-dimer concentration in this horse was measured several times using both assays with similar results, and even dilution curves confirmed the same findings. The reason for this one high value on the Stago assay is not known. However, it seems most likely that in this case the assay was measuring something other than d-dimer. As the assay was developed for humans and built on murine antibodies, it is feasible that there was a form of cross-reaction with another type of matrix antibody present in this horse, potentially due to its clinical condition. We do not know the cause of this finding, which was only seen in one horse, but it highlights the need to combine laboratory results with the individual patient’s clinical manifestations. This horse was thus excluded from this study based on Cook’s distance. The group comparison was performed with and without the outlier. When excluding the outlier, the statistical difference between healthy horses and horses with low SAA disappeared. Both assays were therefore still able to distinguish clinically healthy horses from the high SAA and NSII groups, but were not able to distinguish the different disease groups. It therefore had no effect on the applicability of the two assays.

It is relevant to know whether d-dimer measurements from the Stago and the NycoCard assays can be compared and whether they classify horses in the same way. The Spaghetti plot shows a relatively good agreement between the two methods, particularly for the lower d-dimer concentrations. However, it seems that d-dimer measurements from the NycoCard assay are consistently higher than for the Stago, especially for the higher d-dimer concentrations. This implies that d-dimer measurements from the same horse measured with both assays cannot be compared directly. It does, however, seem that there is good agreement in terms of classifying horses as having a low or high d-dimer concentration, with the exception of three horses with noticeably different results across the two assays. A Passing Bablok regression between the two assays showed that the lower 95% CI for the slope came close to, but did not contain, one. However, the 95% CI for the intercept did contain zero [24]. This combined with a moderately good [28] positive Spearman correlation indicates acceptable coherence between the two assays. A Bland–Altman plot is one of the most commonly used methods to quantify the agreement between two quantitative measurements [29]. The Bland–Altman plot in this study showed a negative bias, which may be the result of average measurements above -2 mg/L (log2). This means that the difference between the two assays is greatest for the higher d-dimer concentrations and that the NycoCard assay measures higher d-dimer concentrations than the Stago assay, as also seen in the spaghetti plot. The best agreement between the two assays is seen for the low d-dimer concentrations. Only two of the measurements are outside the 95% LOA, which is acceptable. Again, this indicates a good agreement between the two assays when it comes to classifying horses with a low or high d-dimer concentration and supports the idea that measurements from the two assays cannot be compared directly.

It has previously been shown that the final concentration of anticoagulant in the blood tube can affect the results of hemostatic markers such as prothrombin time (PT) and activated partial thromboplastin time (aPTT) due to a dilution effect [30–32]. The d-dimer concentration in the two samples from the NSII group that were collected in 3.8% as opposed to 3.2% sodium citrate tubes could thus potentially be diluted. However, it has been shown that d-dimer concentration is unaffected by the final anticoagulant concentration in the sampling tube [33]. The d-dimer concentration for these two samples did not vary markedly from the remaining measurements in the NSII group and it seems likely that the sodium citrate concentration did not significantly influence the d-dimer measurement.

The two assays vary in their ease of use. The Stago machine is an automated assay that can run up to 20 samples at a time. It requires substantial knowledge regarding the setup and handling of the machine, and trained personnel must be present when samples are analyzed. This limits its usefulness in out-of-hours situations. However, once the samples are loaded and running, the machine does the remaining work, which means that a large quantity of samples can easily be analyzed in a short amount of time. Therefore, this assay might be useful in a hospital setting where a large number of samples are run each week or for research purposes. In contrast, the NycoCard assay is an easy-to-use manual tabletop assay. Each test disc is packed separately, and it is thus highly applicable for testing individual animals, even in emergency situations.

In this study, horses were grouped according to being clinically healthy, the degree of systemic inflammation based on the SAA concentration at admission to the hospital or a confirmed diagnosis of thrombotic GI disease caused by *S. vulgaris* (also called NSII). Some of the horses in the low SAA group were diagnosed with an inflammatory disease process after a few days at the hospital, and the low SAA group and high SAA group therefore both included similar types of disease processes but of different duration or severity.

A limitation of the study is the relatively small number of horses in each group for the group comparison. However, it was still possible to observe a significant difference between the clinically healthy horses and the high SAA and NSII groups. Identifying differences between the different disease groups would require around 200–300 horses, which would not be realistic or ethically justifiable when it might not be relevant to the daily clinical work. In addition, the investigated assays should ideally be compared to an established gold standard to calculate a cut off value for thrombosis and assess the true diagnostic sensitivity and specificity for both assays. However, as no gold standard is available in equine medicine, and as it was not possible to perform a postmortem examination of all horses, this was not possible.

## Conclusion

The Stago assay was found to be reliable for measuring d-dimer in equine PPP based on intra- and inter-CVs and linearity under dilution. It was able to distinguish horses in the high SAA group and the NSII group from clinically healthy horses. The assay is considered only moderately user friendly and may not be an appropriate stall-side test, but can be used for research purposes and in larger clinics or hospital facilities.

The NycoCard assay was close to the accepted limit with regard to the medium pool for the intra- and inter-CVs. The assay was further from the accepted limit for the low pool. However, as these values are considered to be below the clinically relevant concentration, the assay is still clinically valuable. The assay was deemed to be reliable based on the linearity under dilution, and it was able to distinguish horses in the high SAA group and NSII group from clinically healthy horses. The assay is quick and easy to use and thus might prove to be a useful stall-side test for detecting critically ill horses in the early stages of disease.

Although the d-dimer measurements from the two assays cannot be compared directly, there does seem to be a good agreement between the two assays in terms of classifying horses as having low or high d-dimer concentration. The agreement seems to be best for the lower d-dimer concentrations. The two assays can thus both be used in a clinical setting to evaluate whether a horse has a low or high d-dimer concentration, but if serial measurements are required, the same assay should be used throughout.

## Methods

This study was approved by the ethical review board of the Department of Clinical Veterinary Science at the University of Copenhagen, and relevant guidelines and regulations were followed. All samples were obtained from December 2017 through to December 2018 and analyzed at the laboratory at the Large Animal Teaching hospital and at the Veterinary Diagnostic Laboratory of the University of Copenhagen, Denmark. Blood samples were collected from clinically healthy horses owned by the University for teaching purposes and following the experimental animal licensing given for the horses. Blood samples from diseased horses were collected from client-owned horses and were collected as part of the diagnostic work-up and with the owner’s consent. All procedures were carried out in accordance with the ARRIVE guidelines.

### Horses

Four groups of horses were included in the study. *1)* Clinically healthy horses from The Large Animal Teaching Hospital at The University of Copenhagen (clinically healthy group). This group included adult horses (> one year of age) deemed healthy based on clinical examination (heart rate, respiration rate, rectal temperature, mucous membrane color, and borborygmia), complete blood count (CBC), and serum biochemistry profiles (including lactate and the acute phase proteins serum amyloid A (SAA) and fibrinogen concentrations) within normal reference intervals. *2)* Horses with GI disease and a mild systemic inflammatory response with a SAA between 30–100 mg/L at admission (low SAA group), *3)* horses with GI disease and a strong systemic inflammatory response with a SAA > 1000 mg/L at admission (high SAA group). Groups two and three included adult horses (> one year of age). *4)* Horses with thrombotic GI diseasecaused by *S. vulgaris* (also called NSII) (NSII group). This group included adult horses (> one year of age) with NSII confirmed at surgery or necropsy, with an area of localized intestinal infarction and without signs of strangulation or enterocolitis. In addition, signs of migrating *S. vulgaris* larvae, seen as thrombosis and arteritis were observed in the cranial mesenteric artery and/or its branches at post-mortem examination or on histology of the resected intestine from surviving horses as has been described previously [34]. The last three groups were all patients referred to the Large Animal Teaching Hospital at the University of Copenhagen. Citrated platelet poor plasma (PPP) from the low and high SAA groups were used in the intra- and inter- assay coefficients of variation (CV) studies and the linearity under dilution. Citrated PPP from the low and high SAA group and the NSII group were used in the group comparison and for the method agreement.

### Samples

As previously described [35] blood samples were collected by clean venipuncture of the jugular vein using a vacutainer system with a 21 G needle on admission to the hospital, at the same time as routine blood samples (including samples for SAA measurement). Four 3.2% sodium citrate tubes (BD, Franklin Lakes, NJ) were filled according to the manufacturer’s recommendation. Samples from two of the horses in the NSII group were drawn in 3.8% rather than 3.2% sodium citrate tubes, but were otherwise handled in the same way. The first tube was discarded. The remaining samples were inverted 4–6 times and then centrifuged within a maximum of four hours at 2,000 g for 15 min. at room temperature [36]. Citrated PPP was then immediately aliquoted into cryotubes and stored for a maximum of 15 months at -80 °C until further analysis. Before analysis, all samples were thawed for approximately 4 min. at 37 °C in a water bath and divided into additional aliquots of low, medium and high pools and then frozen again at -80 °C. When ready for analysis, these aliquots were thawed in the same manner as mentioned above and then vortexed. All samples therefore underwent two identical freeze–thaw cycles, which should not significantly influence the d-dimer concentration [37].

Pools with low, medium, and high d-dimer concentrations were created based on prior analysis carried out on the Stago assay on the horses from the above-mentioned groups. Horses were categorized in such a way as to achieve the desired d-dimer concentrations: A) the low d-dimer pool (*n* = 4 horses) with an average final d-dimer concentration of 0.23 mg/L, B) the medium d-dimer pool (*n* = 3 horses) with an average final d-dimer concentration of 0.52 mg/L, and C) the high d-dimer pool (*n* = 3 horses) with an average final d-dimer concentration of 0.72 mg/L.

### Stago assay

The STAGO STA-Liatest D-di + (Stago) assay (Trioloab, Brøndby, Denmark) is a fully automated humane immunoturbidimetric assay. It performs a photometric measurement of the change in turbidity in a suspension of sample material and reagent containing micro particles. These micro latex particles are covered with two murine monoclonal antibodies with a high specificity for human d-dimer. The binding of antibodies with d-dimer leads to an agglutination of the micro latex particles, and thus a change in turbidity depending on the d-dimer concentration in the sample. The assay provides a quantitative determination of d-dimer in mg/L fibrinogen equivalent units (FEU), the true mg/L d-dimer concentration is considered approximately half the FEU value. The assay has a preset measuring range of 0.27–20 mg/L FEU (0.13–10 mg/L d-dimer) based on human d-dimer samples, and the machine is set to automatically dilute samples above 4.0 mg/L FEU. The measuring interval is 0.01 mg/L [38].

### NycoCard assay

The NycoCard™ D-dimer assay (“NycoCard”) (Abbott Laboratories, Copenhagen, Denmark) [39], is based on an immunometric flow through principle, with plasma d-dimer molecules being trapped on a surface membrane with d-dimer specific monocloncal antibodies. The addition of a secondary labelled antibody generates a subsequent color development, with the color intensity being proportional to the d-dimer concentration. The assay has a measuring range of 0.1–20 mg/L, a calibrated human assay range of 0.1–10.0 mg/L and a measuring interval of 0.1 mg/L (NycoCardTM D-dimer, technical support, Abbott Laboratories, Copenhagen, Denmark) [40]. Measurements displayed by the machine as < 0.1 mg/L in this study were converted to 0.09 mg/L for statistical purposes.

### Test evaluation

Imprecision was evaluated in both tests by routine descriptive analytical procedures. Intra- and inter-CVs were examined by running the low and medium d-dimer pools in duplicate ten times in one day and then once daily for ten consecutive days with an imprecision performance acceptance level of 5% for automated assays (Stago) and 10% for manual assays (NycoCard) [24].

Linearity under dilution was used to determine inaccuracy for both assays by analyzing the high d-dimer pool both undiluted and diluted with 0.9% saline to obtain the following concentrations of d-dimer: 100%, 80%, 60%, 40%, 20% and 0%. A Passing Bablok regression was used for both tests. Linearity performance was considered acceptable if the confidence interval of the slope and Y-intercept included 1 and 0, respectively.

Group comparisons of the assays were evaluated by comparing the median and interquartile range across the four groups of horses (clinically healthy, low SAA, high SAA, and NSII) for 32 horses. Data did not follow a normal distribution and applying various log-transformations did not improve this, so a Kruskal Wallis test was used. A Dunn’s multiple comparisons test was used for post hoc analysis.

### Method comparison

A method comparison was performed examining d-dimer concentrations using the Stago and NycoCard assays in parallel across the four groups of horses. The agreement between the two assays was illustrated in a spaghetti plot, with a Passing Bablok regression, a Spearman correlation test, and a Bland Altman plot on log2 transformed data, which brought the difference between the two assays closer to a normal distribution than with the original data. The Spearman correlation was considered poor if the r-squared value was ≤  ± 0.2, fair with a value >  ± 0.2 to ≤  ± 0.5, moderate with a value >  ± 0.5 to ≤  ± 0.7 and strong with a value ≥  ± 0.8 [28].

Statistical analyzes were performed using Microsoft Excel 2016 (Washington, USA), R Studio (Boston, MA, USA) and GraphPad Prism 8.1.0 (San Diego, USA). Data was assessed for normality using graphic evaluation. A significance level of < 0.05% was set for all analyzes.

The complete dataset was examined for extreme measurements using graphic representation and Cook’s distance (= 4/n). If a variable was found to be an extreme outlier with a strong influence on the statistical analysis, it was excluded. Any exclusions was accounted for in the results paragraph.

## Supplementary Information

**Additional file 1.**

**Additional file 2.**

## Data Availability

If deemed relevant or is of interest raw data can be submitted.

## Abbreviations

aPTT: Activated partial thromboplastin time
BW: Body weight
CBC: Complete blood count
CI: Confidence interval
CV: Coefficients of variation
GI: Gastrointestinal
LOA: Limits of agreement
NSII: Non-strangulating intestinal infarction
NycoCard: NycoCard™ D-dimer assay
PPP: Platelet poor plasma
PT: Prothrombin time
Q_1_: 1^st^ quartile
Q_3_: 3^rd^ quartile
SAA: Serum amyloid A
Stago: STAGO STA-Liatest D-di + assay
*S. vulgaris*: *Strongylus vulgaris*
